# Kawasaki Disease: The Role of Immune Complexes Revisited

**DOI:** 10.3389/fimmu.2019.01156

**Published:** 2019-06-12

**Authors:** Stephanie Menikou, Paul R. Langford, Michael Levin

**Affiliations:** Section of Paediatrics, Division of Infectious Diseases, Department of Medicine, Imperial College London, London, United Kingdom

**Keywords:** Kawasaki disease, immune complexes, immunopathology, IVIG, pathogens

## Abstract

Kawasaki disease (KD) is an inflammatory disease in children associated with vasculitis affecting predominantly the coronary arteries and is now the most common cause of acquired heart disease in children in developed countries. The etiology of KD is unknown but epidemiological studies implicate an infectious agent or toxin, which causes disease in genetically predisposed individuals. The presence of immune complexes (ICs) in the serum of children with KD was established in numerous studies during the 1970s and 80s. More recent genetic studies have identified variation in Fcγ receptors and genes controlling immunoglobulin production associated with KD. In this review we link the genetic findings and IC studies and suggest a key role for their interaction in pathophysiology of the disease.

## Introduction

Kawasaki disease (KD) is a pediatric inflammatory disease associated with a self-limited vasculitis. It was first described in Japan by Dr. Tomisaku Kawasaki in 1967 and has subsequently been recognized as an important childhood disorder worldwide (1). The cause of KD remains unknown, but the seasonal occurrence, prevalence in young children, infrequent relapse, and occurrence of epidemics all point to an infectious agent as the cause. The disease is diagnosed based on the characteristic constellation of fever, lymphadenopathy, rash, mucous membrane changes, and desquamation associated with laboratory evidence of inflammation. In the absence of specific treatment the clinical symptoms usually evolve over 7 to 14 days and resolve gradually, but the fever and severe inflammatory process might persist for 2 to 4 weeks in severe untreated cases (2).

The clinical and epidemiological features of KD strongly suggest an infectious etiology, including the occurrence of epidemics, seasonal variation in incidence and clustering of cases. Although many pathogens have been implicated via superantigen toxins, staphylococcal and streptococcal toxic shock syndromes, other studies have failed to confirm these findings (3–6). A range of other bacteria including *Yersinia pseudotuberculosis* (7–9)*, Propionibacterium acnes* (10), *Mycoplasma pneumoniae* (11, 12), *Chlamydia pneumoniae* (13), *Rickettsia* species (14), and *Coxiella burnetii* (15) have been reported. Also, numerous viruses including Epstein Barr virus (16), retroviruses (17, 18), adenovirus (19), and measles virus (20) have been reported to be associated with KD. None of these reported viruses or bacteria have been convincingly replicated.

Intravenous immunoglobulin (IVIG) was shown to be an effective treatment in the 1980s, and the majority of children with KD show a rapid resolution of fever and symptoms following infusion of IVIG (2, 21, 22). IVIG treatment greatly reduces the risk of coronary artery aneurysm (CAA) but 5-20% of KD cases fail to respond, have persistence or reoccurrence of fever and require additional anti-inflammatory treatments (22, 23). IVIG-resistant patients have an increased risk of developing CAA (23–25). For patients that are unresponsive to initial IVIG treatment, a range of alternative therapies to reduce inflammation have been advocated including steroids, and treatment with tumor necrosis factor (TNF) inhibitors. There is growing evidence from recent studies that other anti-inflammatory agents, including cyclosporine A (CyA) and anakinra [an interleukin 1 receptor antagonist (IL1-RA)] may be beneficial in reducing coronary artery damage and at the same time controlling the inflammatory process in KD patients (22, 26–29).

## Immunopathogenesis in KD

A central feature of KD is the activation of the immune system (2). The immune response in the acute phase of patients with KD involves activation of many different components of the innate and adaptive immune systems (30). There is an intense inflammatory response in the initial weeks of the illness, with elevation in white blood cell (WBC) counts and activation of most classes of WBCs including neutrophil leucocytosis and elevated eosinophil counts in acute and subacute KD patients (31). There is also an intense inflammatory response in the initial weeks of illness with elevation of acute phase proteins such as C-reactive protein (CRP), procalcitonin (PCT) (32), erythrocyte sedimentation rate (ESR) and higher values of alanine aminotransferase and γ glutamyl transferase (GGT) (33). In a study by Katayama et al. (34) peripheral blood CD14^+^CD16^+^ monocytes were also increased during the acute phase of KD.

Immune cells including monocytes/macrophages, and T and B lymphocytes produce cytokines such as interleukins (IL) (35). In KD, many cytokines are expressed at significantly higher levels than normal during the acute phase (2). Gene expression profile studies by Hoang et al. (36) identified increased transcript abundance of the genes *IL-1R, IL1RN, IL-1RAP*, and *IL-1R2*, supporting the importance of the IL-1 pathway in the pathogenesis of KD (36). Other cytokine genes up-regulated in the blood of KD patients, compared to children with other febrile illnesses and healthy children-adult volunteers, include *IL-6* (35), *IL-8* (2), and *IL-17A* (37). A study by Rowley et al. (38) gave another insight into the immunopathogenesis of KD as the authors found significantly increased levels of IgA plasma cells in the trachea, kidney, coronary artery and pancreas of acute phase patients who died; supporting the entrance of a pathological agent through the upper respiratory tract.

Besides the blood changes in neutrophils, monocytes, lymphocytes and cytokines, changes occur in the tissues of acute KD patients as well. Neutrophils are predominant in the peripheral blood of the acute KD patient and have been identified in the arterial wall early in the disease (30, 39, 40). Immunohistochemical studies on CAA from fatal cases who died in the acute and subacute phases of disease showed that invasion of neutrophils into the arterial wall is followed not only by monocytes/macrophages but also by dendritic cells (DCs) and lymphocytes (30, 41). Coronary arteritis in acute KD patients begins with infiltration of a small number of macrophages, lymphocytes and neutrophils in both tunica adventitia and intima and not in the media of the artery. In the subacute phase of illness, the internal elastic lamina is disrupted and there is inflammation of all the layers of coronary artery due to infiltration of lymphocytes, neutrophils and macrophages into the arterial wall (42). The main lymphocyte population identified in the coronary arterial lesions of KD patients were cytotoxic (CD8^+^) T cells (43, 44). Additional studies confirmed that CD8^+^ T cells were abnormally activated in the acute phase of KD with an imbalance between their activation and inhibitory actions (43). The results strongly suggest that in the acute phase of KD there is an antigen-driven immune response (41). Yilmaz et al. (45) found that there were increased numbers of mature myeloid DCs with high human leukocyte antigen-antigen D related (HLA-DR) expression in coronary artery lesions of four patients, thus further supporting the antigen-driven immune response as DCs process and transport antigens to the T cell's surface (39, 46).

## Role of Immune Complexes

Antigen-antibody complexes are formed when antibodies are produced against a circulating or tissue antigen. The antigen may be exogenous from an infectious agent, toxin or drug, or may be endogenous as occurs in autoimmune disorders. ICs are formed during many infectious and inflammatory diseases, and may play an important role in immunopathogenesis of infectious and inflammatory processes. They are normally taken up by inflammatory cells through binding of the heavy chain constant region to immunoglobulin Fc receptors (FcRs). Binding of immunoglobulin to some classes of FcRs (FcγRI, FcγRIIA, FcγRIIC, FcγRIIIA, FcγRIIIB) may lead to activation of inflammatory cells while binding to FcγRIIB result in suppression of inflammation. ICs may bind to FcRs and activate a range of cells including monocytes, basophils, eosinophils, lymphocytes and neutrophils (47). The interaction of ICs with neutrophils can lead to phagocytosis, degranulation and respiratory burst generation. In addition, ICs can bind to platelets directly as well by the recognition of FcRs on platelet membranes (47). Platelets are multifunctional cells that contribute to both inflammatory and coagulation functions. Their granules contain a broad range of inflammatory substances including anti-microbial proteins, such as defensins and kinocidins, which may contribute to internalizing pathogens to promote their clearing from host tissues and the bloodstream (48). ICs can have a significant role in immune responses by inducing inflammatory processes and eliminating antigens (49). ICs may also precipitate in tissues, leading to local inflammation or organ damage, complement activation and influx of inflammatory cells (50). They are particularly important in renal diseases such as membranoproliferative glomerulonephritis and systemic lupus erythematous (SLE) nephritis. ICs have been implicated in many autoimmune disease processes including rheumatoid arthritis (RA) and SLE where their deposition in tissues contributes to organ damage (50). They are also found circulating in the blood in patients with malignancies and many infectious diseases such as subacute sclerosing panencephalitis, hepatitis and dengue hemorrhagic fever (50, 51).

ICs attracted attention as possible components of the inflammatory process in KD because of their well-established role in experimental and clinical forms of vasculitis. In the serum sickness model of IC vasculitis, following injection of a foreign antigen and production of antibody, ICs form in the circulation in the second and third week following exposure, as antibody production against the foreign antigen occurs, and contributes to tissue damage through activation of platelets and other inflammatory cells (52). As already mentioned, ICs have important roles in many immunological diseases including SLE, RA, and many forms of nephritis and vasculitis. It was therefore not surprising that a role for ICs was considered in KD.

## Immune Complexes in KD

**Figure 1:**
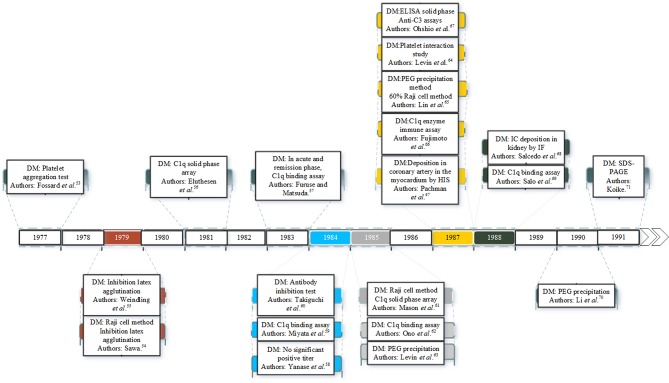
Studies of IC detection in KD patients. DM is the detection method.

There is considerable evidence that ICs are involved in the pathogenesis of KD. Since the 1970s many studies have identified ICs in the plasma or serum of KD patients as shown in Figure 1. ICs were first reported in patients with KD in 1977 (53). Since then many studies have documented ICs in the acute and early convalescent phase of the disease (47, 53–71). The ICs have been identified by a range of different methods including platelet aggregation tests, C1q binding assay, ELISA and polyethylene glycol (PEG) precipitation.

### The Time Course and the Nature of Immune Complexes in KD

Sequential studies of patients in the pre-immunoglobulin therapy era enabled the time course of ICs in the plasma to be identified. ICs appear in the circulation in the first 7 days of the illness, and reach a peak concentration in the second week, before declining slowly by the third and fourth week of the illness (63). The first documented case of ICs in KD in UK was in 1977 in a 22-month-old Japanese boy presenting with increased serum levels of immunoglobulins A and M (IgA, IgM), upper normal levels of immunoglobulin E (IgE), and a strongly positive result on the platelet aggregation test for IC detection (on day 12) (53). Raised concentrations of IgE were also detected in the acute phase of KD patients in both studies of Kusakawa et al. (72) and Furukawa et al. (73). A possible role for IgE was suggested in pathogenesis of vasculitis by increasing vascular permeability (53). In another study, Weindling et al. (55) described a 9 month old Caucasian girl showed normal levels of IgE and complement but increased levels of ICs containing immunoglobulin G. Eluthesen et al. (56) found high levels of circulating ICs in 48 out of 81 early convalescent KD patients (56). Furuse et al. (57), also found high levels of ICs further supporting the possibility of immunopathological involvement in KD.

### Are Immune Complexes Associated With the Severity in KD?

Several studies have confirmed the presence of IC in the blood in the early phases of KD and an association with severity. These include, higher levels of ICs and immunoglobulin G (IgG) being detected in KD patients with CAA in a study by Miyata et al. (59) and that of Takiguchi et al. (60) who found ICs using the anti-antibody inhibition test in 23% of the patients. Mason et al. (61) detected ICs in the sera of 69% KD patients in all stages of illness but found no correlation between CAA development and the presence of ICs.

In contrast, Ono et al. (62) found that the group of KD patients with CAA had higher levels of ICs compared to the group that did not develop CAA. IgG ICs were present in 47.1% of KD patients in a study by Li et al. (70). The latter authors also found that two of the human IgG subclasses, IgG1 and IgG3, were predominant in ICs and significantly higher in patient sera (70). Levin et al. (63) documented the presence of circulating ICs in the serum of 19 children with KD in the second and third weeks of the illness, with the severity of disease being associated with an increased concentration of ICs (63).

Further studies are necessary to determine whether there is a causal link between ICs and severity of KD. For example, advances in proteomic analytical techniques could be used to characterize the composition of ICs in patients with different degrees of severity of KD and those in children with other illnesses. Such experiments would have the added value of potentially identifying any causative agent of KD.

### Potential Role of Immune Complexes in KD

Levin et al. (63) suggested the hypothesis that KD has three distinct pathophysiological phases: during the first phase (acute) there are high levels of antigen (arising from the unknown pathogen) in the blood and patients exhibit fever, lymphadenopathy and mucocutaneous manifestations (63). In the second phase (days 7–21), ICs are detected in the serum and bind to inhibitory and activating FcRs expressed by macrophages, DCs, B cells, neutrophils and platelets. FcRs may be important as the mediators between ICs and cellular responses. Furthermore, ICs may deposit in the tissues and activate complement, contributing to tissue damage. In the final stage, ICs are cleared and there are persisting antibodies against the causative antigen.

ICs develop when antibodies form complexes with their unknown antigens in the circulation. These complexes interact with platelets causing aggregation and release of vasoactive mediators such as histamine and serotonin, an important part in the initiation of vascular damage (63). Serotonin is an important vasoactive agent stored in the dense granules (74, 75). Levin et al. (63) showed that ICs from KD patients triggered release of serotonin from platelets. Recent studies consider serotonin a critical platelet component implicated in mediating vasodilatation and neutrophil activation (74).

Activated platelets recruit leukocytes and neutrophils to the affected sites following an infection or tissue damage (74). The binding of platelets to neutrophils occurs by CD62P ligand via CD162 which is a P-selectin glycoprotein ligand 1 (48). DCs are also activated by platelets. DCs role is to present antigens to B and T cells (48). It is suggested that naturally occurring autoantibodies or ICs are sufficient to activate DCs (76). This is accomplished by the binding of ICs to FcRs on DCs leading to phagocytosis and antigenic peptide presentation on MHC class I and II molecules that are recognized by T cells via cluster of differentiation 40 (CD40) and CD40 ligand (CD40L) which is also expressed by human platelets. Ultimately, the continuation of antibody production and phagocytosis leads to the elimination of the antigen.

**Figure 2:**
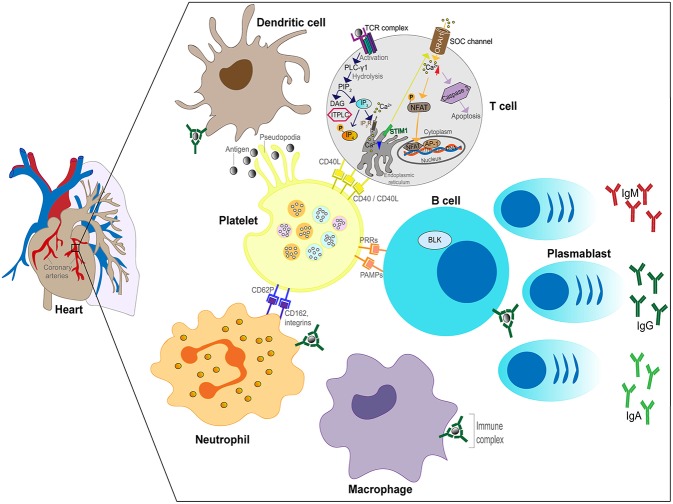
Schematic of the role of ICs in the inflammatory process in KD. Plasmablasts produce antibodies (IgG, IgM, or IgA) against the unknown pathogen resulting in IC formation. ICs bind to FcRs expressed by neutrophils, B cells, DCs and macrophages leading to activation of the cells. Neutrophils invade the arterial wall followed by macrophages, DCs and T cells. ICs activate platelets releasing soluble mediators and binding to neutrophils. Phagocytosis of ICs by macrophages leads to antigenic peptide presentation and T cell activation.

In the third phase (convalescent), the ICs disappear from the circulation and high levels of antibody against the pathogen are present in the blood, (63) coinciding with defervescence of fever and subsidence of mucocutaneous manifestations. Vascular damage that was initiated in the second week of illness can lead to myocardial infarction and CAA that are most commonly detected after this phase of the illness. In summary, the implication of the Levin et al. (63) study was that KD has different phases involving different mechanisms (feverish phase driven by an unknown pathogen and antigen excess, IC vasculitis and convalescent phase after clearance of both antigen and ICs). The proposed IC involvement in KD is illustrated in Figure 2.

Although there are many studies demonstrating ICs in KD patients, a few have not supported an important role for ICs. In the study of Mason et al. (77) no correlation was found between the symptoms of KD (in particular fever and CAA) and the presence of ICs. In another study by Falcini et al. (78), 66% of KD patients had circulating ICs, with lower levels in convalescent compared to acute phase sera, but there was no significant relationship between ICs and the occurrence of aneurysm or any other clinical symptoms.

It should again be noted that most of the literature on ICs in KD dates from the 1980s, and most of the reports give insufficient clinical details to accurately determine the stage of the disease at which blood was tested for ICs. Furthermore, a range of different assays were used to detect ICs, some based on interaction with specific cells (such as platelets or Raji cells), and others based on the complement binding property of ICs. The fact that not all studies have confirmed ICs in all KD patients may reflect the differences in timing of the test in relationship to disease onset, or the differences in methodology for IC detection. Since the introduction of treatment with IVIG, studies on the “natural” pathophysiology have become difficult, as the IVIG may work in KD through an effect on the ICs or interaction with their receptors. Subsequent studies in the field were focused on macrophages, B cells, cytokines and T cells, and did not address a causal link between ICs and KD severity. It should be noted that these attempts to link severity (usually based on presence or not of CAA) and ICs were undertaken in the early days of ultrasound detection of CAA, and before the introduction of Z scores to quantify the size of CAA. To fully address the question of a relationship between presence of ICs and disease severity, more detailed studies using, in addition to quantitative documentation of coronary artery dimensions by echocardiography, other markers of disease severity such as height or duration of inflammatory markers such as CRP, IL-6, white cell or platelet count as well as response to IVIG would be needed. Furthermore, while the ICs may themselves contribute to disease pathogenesis, the nature and load of antigen may also be important contributors to disease severity. Severity of the initial febrile illness may be caused by a direct effect of the infecting agent or its antigens or toxins. The production of antibodies (the rate and nature of which may be genetically determined) and formation of ICs may be a second factor determining severity, and how ICs interact with cellular receptors such as the Fc receptors on immune cells or other tissues (which also depends on genetic factors) may play an additional role.

## Methods to Detect Immune Complexes

A variety of methods are used for IC isolation. Some of these methods are based on the attachment of ICs to specific cellular receptors such as Complement component 3 (C3) receptors on Raji cells, platelets or FcRs on macrophages or C1q (50). However, these techniques identify elements that are associated with but do not directly identify ICs themselves (79). Morrow et al. (80) found that PEG precipitation can be used to isolate ICs, and that there was no correlation between serum IgM and IgG levels and PEG-precipitated IgM and IgG levels in ICs in disease sera. Other studies supported Morrow's findings, and that lower concentrations of PEG resulted in higher yields of ICs with minimal free IgG or other proteins being precipitated (79). PEG appears to precipitate antibody preferentially when in complexes with antigens. PEG causes precipitation of proteins and ICs by steric exclusion in which larger proteins are precipitated only from the regions of the sample that are surrounded by linear chains of PEG (81, 82). Steric exclusion increases concentration and when the solubility is exceeded, precipitation of proteins occurs (82).

Several other techniques have been used to detect ICs including platelet aggregation tests for circulating ICs (83, 84), gel diffusion methods which measure bound exogenous C1q to ICs and aggregated y-globulin (85), anti-complementary activity by binding to Complement 1 (C1) (86, 87), gel filtration using Sepharose 6B (88), or using radiolabeled C1q that reacts with sera containing ICs in combination with PEG precipitation for removal of free C1q (89). Additional studies used labeled macrophages and aggregated IgG to study IC inhibition of phagocytosis (79). Other techniques for IC quantification use binding to C1q followed by addition of a radiolabelled anti-human IgG to determine the amount of ICs (90), or using purified bovine conglutinin (instead of C1q) to detect ICs with minimal monomeric IgG influence (91).

As indicated above, many different methods have been used to detect ICs. However, those involving PEG precipitation are considered the most reliable due to their simplicity, involving only comparatively few technical steps, that they do not induce IC formation (92), and there is low propensity for denaturation of proteins present, facilitating the search for new antigens within ICs. It should be noted that, dependent on the concentration used, PEG-precipitated sera may contain free immunoglobulins and C3 but also other proteins such as albumin and fibronectin (92, 93), which can be advantageous when such proteins are also under investigation. The last decade has seen an explosion in mass spectrometric-based proteomic techniques (94, 95), and we believe that their use with PEG-precipitated ICs from KD patients has great potential to identify the cause of the disease, complementing genetic approaches (as reviewed below).

## Genetic Studies Support a Role of Immune Complexes in KD

Although all ethnic groups are affected by KD, the high incidence in Asian countries, and in those of Asian ethnicity that migrate to other countries (96), and the higher frequencies among siblings and twins, suggests a genetic contribution to the risk of KD (97, 98).

Candidate gene case control studies have identified a large number of associations with KD susceptibility. These include polymorphisms in cytokines such as *IL1-RA* (99), *IL-4* (100), *IL-10, TGFB2* and *TGFBR2* (genes of the TGF-β pathway) (101) and chemokines including Chemokine (C-X-C motif) receptor 1 and 2 (102) and Chemokine (C-C) receptor 2, 3 and 5 (102), matrix metallopeptidases such as matrix metallopeptidase 3 (*MMP3*) (103), and HLA genes (*B35, B37* and *Bw44*) (104). Polymorphisms in HLA, transforming growth factor beta (TGF-β) and calcium signaling pathways account for a proportion of the genetic risk (30). Genes associated with increased risk of CAA have also been identified in association studies including a polymorphism in the *IL-10* promoter (105), a functional polymorphism in the *MMP-13* gene (103), a polymorphism of the inflammatory gene *CRP* (106) and the *TNF-*α gene (106).

Genome wide association studies (GWAS) have identified a number of validated associations including a functional single nucleotide polymorphism (SNP) in the *ITPKC* gene, encoding inositol 1,4,5-trisphosphate 3-kinase C, whose role is to negatively regulate T cell activation through the calcium signaling pathway (Figure 2) (107). Functional polymorphisms in *ITPKC* can result in increased T cell activation, increased expression of cytokines, and these polymorphisms were found to be significantly associated with increased susceptibility to KD and an increased risk of coronary artery lesions in US and Japanese children (107).

Further genetic associations with KD susceptibility published to date suggest an important role for ICs in KD. Firstly, the finding from GWAS that polymorphisms in the IgG receptor gene *FCGR2A*, identified as a susceptibility locus for KD in European and Asian populations, is an important clue to the role of ICs (108). Immune cells (DCs, neutrophils, monocytes/macrophages) express on their surface FCGR2A and when this receptor is ligated to ICs it transduces activation signals in the cells (Figure 2) (109). These signals lead to either activation of inflammation or in some cases immune suppression. Therefore, polymorphisms in *FCGR2A* suggest an important role for IgG receptors in the immunopathogenesis of KD as individuals who carry the functional polymorphism may respond to ICs differently from the majority of the population with a persistent or excessive inflammatory response.

Another genetic association suggesting the importance of ICs in KD is the association with polymorphisms in CD40 and CD40L. CD40 is expressed on the endothelial cells, epithelial, B-cells, DCs, monocytes, macrophages, fibroblasts, myofibroblasts and it engages with CD40L (110). This ligand is a type II integral membrane protein of the TNF family that is expressed primarily on the surface of activated CD4+ T cells, B cells and platelets transducing signals that result in endothelial cell damage, various inflammatory and immune responses (111). CD40/CD40L engagement on the surface of DCs enables cross-presentation of antigens and promotes cytokine production to effectively stimulate activation and differentiation of T cells (110). Also, their interaction on B cells promotes early immunoglobulin production, isotype switching and enhancing affinity for antigen by somatic hypermutation of immunoglobulin genes (110). Acute KD patients had significantly higher expression levels of CD40L when compared to febrile controls, and these expression levels were higher in KD patients with CAA (111, 112). Gene polymorphisms in *CD40* (113, 114)/*CD40L* (115) are associated with KD susceptibility and CAA formation. These genetic polymorphisms and differences in expression levels of CD40/CD40L might affect immunoglobulin production rate, or somatic hypermutation of immunoglobulin that would enhance affinity for antigen or lead to different isotype switching. Hence, individuals who are genetically determined to produce a different class or amount of immunoglobulin antibodies may produce different types of ICs.

Besides *FCGR2A* and *CD40/CD40L*, the association of the *BLK* gene with susceptibility to KD further supports the hypothesis that a genetically determined difference in rate or type of antibody produced in response to an infection is involved in KD. *BLK* is a gene encoding B-lymphoid tyrosine kinase, expressed primarily in the B cell lineage and is part of one of the members of the Src family of kinases downstream of the B cell receptor. B cell receptor signaling is important for secretion of antibodies and B cell activation. An important example of B cell negative regulation, is through the CD40/CD40L pathway to repress T cell proliferation (116). GWAS showed that the most significant association of the *BLK* gene with KD was in individuals carrying the risk T allele of rs273640 in the promoter region and the first intron of the *BLK* gene (8p23.1) (117). During the acute phase of KD, this risk allele is associated with lower expression of *BLK* in peripheral blood B cells altering B cell function (116). One consequence might be impaired or abnormal antibody production. Another genetic variant in *BLK* region, rs2254546 at an intergenic region between *FAM167A* and *BLK* genes, was significantly associated with KD in the Japanese population (113).

Thus, both *CD40/CD40L* and *BLK* genetic variant associations may result in KD patients having a different immunoglobulin production speed, quantity or immunoglobulin isotype classes and therefore differing from healthy children in types of ICs formed. Also, the differences in FcγRs due to functional polymorphisms in the IgG receptor gene *FCGR2A*, contribute to an aberrant response in which ICs are central players.

## How Might IVIG Effect an IC-Mediated Inflammatory Process?

In view of the evidence of aberration in KD patients in genes involved in the production of immunoglobulin and class switching, or clearance of ICs, the dramatic effect of IVIG may be due to several possible effects on IC-mediated inflammation. IVIG administration results in rapid resolution of symptoms and reduction in rash, fever and conjunctival injection in the majority of patients. There are several possible mechanisms by which IVIG might induce such a rapid resolution of inflammation in KD. Antibodies in IVIG might neutralize the unknown toxin or antigen that triggers the inflammatory process. However, an alternative explanation is that antibodies in IVIG might compete with endogenous antibody, to alter the size or composition of the ICs. Alternatively, the immunoglobulin might compete with the ICs for binding to FcγRs, thus reducing the aberrant cellular activation occurring in KD. In a more generic view, IVIG treatment may depend on the F(ab′)2 portion of immunoglobulins which mediates inhibition of the antibodies, cytokines and receptors. Alternatively, it may depend on the Fc portion which is associated with inhibition of interaction with complement, regulation of DCs, B cells and FcγR binding and activation. Attempts to define the mechanisms of IVIG action in other diseases may be relevant to KD. Debre et al. (118) found that in acute immune thrombocytopenic purpura (ITP), purified Fc fragments alone were an effective treatment.

Nagelkerke et al. (119) found that IVIG had an inhibiting effect on FcγRs from monocyte-derived macrophages and this blocking was most effective in the presence of dimeric and multimeric IgGs. Preference toward dimeric/multimeric IgGs could be linked to the fact that most FcγRs can only interact with antibodies in the form of ICs. Abe et al. (120) found monocytes and macrophages were suppressed following IVIG infusion due to down-regulation of FcγRIA and FcγR3A. These studies suggest that IVIG may compete with ICs for binding to the FcγRs, preventing activation of these cells by ICs.

IVIG may also act through DCs which are involved in antigen capture and presentation of cell-bound antigens to lymphocytes, initiating T cell responses. IVIG modulates DC functions and is associated with induction of regulatory T cells (Treg) via cyclooxygenase (COX)-2-depedent prostaglandin E_2_ (PGE_2_) (121). This effect was Fab' dependent, and affected by sialylation of immunoglobulin. The importance of Treg's is highlighted by the observation that Tregs stimulated via Fc secrete IL-10 (122), and IL-10 levels were significantly higher in KD patients with CAA and in IVIG non-responders before IVIG treatment (123). IC bind to Fc receptors enabling priming of CD8 cytotoxic T cell responses and expansion of Treg cells. ICs are more active in triggering DC activation than antigen alone and trigger stronger immune responses (109). We hypothesize that IVIG infusion in KD inhibits binding of ICs to the DCs, reducing immunological responses.

There is also evidence that IVIG affects Treg secretion of anti-inflammatory cytokines such as TGF-β and IL-10 (124). Immunoglobulin can induce Treg production and function by promoting STAT5 expression. CD8+ T cells are also abnormally activated during acute KD, and the greater the degree of activation the higher the chance of IVIG resistance (43).

Neutrophil activation is also affected by IVIG. Sialic acid-binding immunoglobulin-like lectin 9 (Siglec) is a surface molecule expressed on neutrophils. IVIG contains antibodies against Siglecs that induce cell death (125), which requires only the Fab' portion of the IgG (126). Thus, although neutrophils express Fc receptors, IVIG may affect neutrophil viability through a number of mechanisms in addition to competing with ICs for bind to the FcγRs.

In summary multiple pathways are targeted by IVIG to reduce fever, tissue inflammation, and prevent CAA. However, further studies are required to understand in depth the mechanisms of action of IVIG that could lead to more effective and targeted therapeutic approaches in KD.

## Conclusions

The 50-year-old mystery as to the cause of KD remains to be solved. We still do not know whether it is caused by an infectious organism, a toxin, a chemical substance or other agent. ICs seem to be important in the etiopathogenesis of KD and may provide a clue to the triggering antigen.

The large number of studies which have documented ICs in the plasma or serum of patients with KD suggests that they play an immunopathogenic role in the disease. The fact that many of the genes now established as being associated with KD are those controlling B cells (*BLK*), immunoglobulin class switching (*CD40/CD40L*) or recognition of ICs' (*FCGR2*) support a role for the antibody response and clearance of antibody in KD. As the ICs are likely to contain antigens which have initiated the immune response, identification of the antigen within the ICs may be an important step in identifying the causative agent of KD. Future studies, using modern proteomic methods to isolate ICs in KD, and characterization of the antigen(s) may lead to identification of the causative agent as well as improved understanding of the inflammatory process.

## Author Contributions

SM, PL, and ML: conceptualization. SM: writing—original draft. SM, PL, and ML: writing—reviewing & editing.

### Conflict of Interest Statement

The authors declare that the research was conducted in the absence of any commercial or financial relationships that could be construed as a potential conflict of interest.
